# Cardiovascular Risk Management In Patients With Rheumatoid Arthritis: A Systematic Review

**DOI:** 10.7759/cureus.58409

**Published:** 2024-04-16

**Authors:** Tarundeep Singh, Bangari Laxmiraj, Rachel Chandra Harika Chukka, Tarika Noor

**Affiliations:** 1 Department of Medicine, Government Medical College, Patiala, Patiala, IND; 2 Department of Medicine, Kamineni Institute of Medical Sciences, Hyderabad, IND

**Keywords:** cv risk prevention, cardiovascular risk management, cardiovascular risks, rheumatoid arthritis, cardiovascular disease

## Abstract

Rheumatoid arthritis (RA) is an autoimmune chronic inflammatory joint disease associated with pain, swelling, and morning stiffness. It not only affects the joints but also exhibits many extra-articular manifestations. It is recognized as an independent risk factor for cardiovascular (CV) abnormalities. The possibility of cardiovascular disease (CVD) risk in patients with RA is about twofold higher compared to non-RA individuals. Therefore, early risk assessment and management of risk factors are crucial to reduce the CV morbidity and mortality associated with RA.

This systematic literature review summarizes the data available on the management of CVD risk factors in RA. A total of 61 articles from the most reputable journals published between 2013 and 2023 were reviewed, of which seven papers were selected for in-depth analysis. We tried to eliminate bias using various bias-eliminating tools. This analysis considers the proposed solution for CV risk prevention and management in RA patients.

Optimal control of disease activity and persistent monitoring of other factors responsible for increased CV events in RA patients is the ultimate management of CV abnormalities. This study summarizes the recommendations for the management of CV risk factors in patients with RA.

## Introduction and background

Cardiovascular diseases (CVDs) account for over 50% of premature deaths in patients with rheumatoid arthritis (RA) compared to non-RA patients [1]. RA affects 0.5-1% of adults, with 5-50 per 100,000 new cases annually [2]. The mismatch of thoughts between the rheumatologist and the primary care physicians is one of the reasons for the underdiagnosis and undertreatment of CVDs associated with RA because rheumatologists consider this aspect of care to be the responsibility of primary care providers [3].

RA is characterized by synovitis and systemic inflammation. It occurs more commonly in women and the elderly. If left untreated, RA can significantly reduce the quality of life, causing joint damage, CVD, and other comorbidities. There is a significant rise in autoantibodies, particularly rheumatoid factor (RF) and citrullinated peptides. Genetic factors account for 50% of the risk for the development of RA [3]. Cardiovascular (CV) events occur about 10 years earlier in patients with RA than in the general population, suggesting RA to be an independent risk for ischemic heart disease [4].

The factors responsible for increased CVD risk in RA patients seem to be independent of traditional CV risk factors [4]. Smoking, hypertension, dyslipidemia, body mass index, insulin resistance, genetic factors, and antirheumatic drug-related cardiotoxicity are considered traditional risk factors for the occurrence of CV events in RA [1].

The immune mechanism includes T-cell activation that leads to endothelial dysfunction, a decrease in endothelial progenitor cells, and arterial stiffness. Pathogenic mechanisms include pro-oxidative dyslipidemia, insulin resistance, prothrombotic state, and hyperhomocysteinemia [5].

The European League Against Rheumatism (EULAR) proposed multiplying the Systemic Coronary Risk Evaluation (SCORE) by 1.5 times in RA patients presenting with at least two of the following: (1) disease duration of more than 10 years, (2) RF or anti-cyclic citrullinated peptide, and (3) the presence of extra-articular manifestations [6].

Despite a growing understanding of these mechanisms, an optimal approach for their prevention in the context of RA is unknown. Gaps in basic knowledge, clinical validation, study design, and methodological practices are the key limitations in the evaluation of CV risk in RA patients [7]. This systematic review evaluates available evidence on the management of CVD risk in patients suffering from RA.

## Review

Methodology

**Figure 1:**
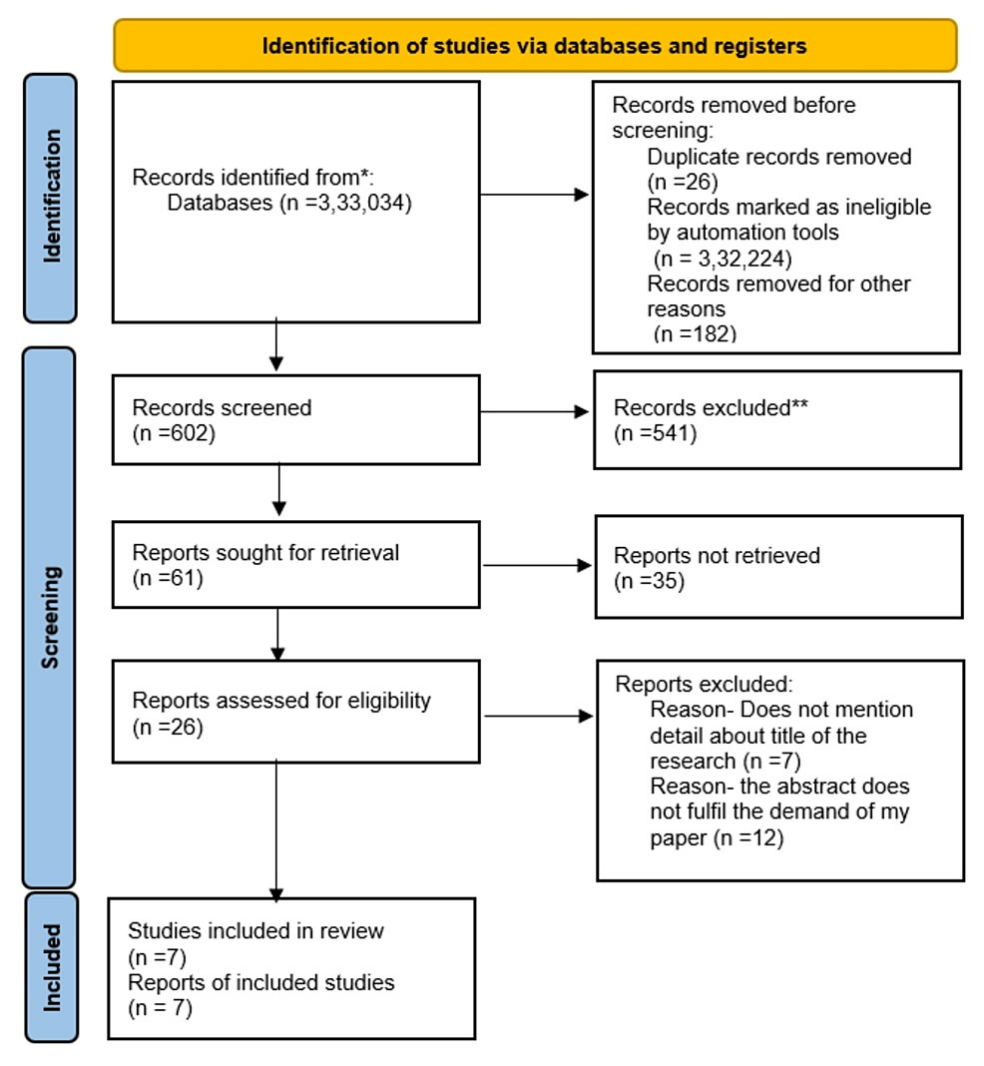
Preferred Reporting Items for Systematic Reviews and Meta-Analyses flow diagram of data inclusion strategy for systematic review.

This review focuses on CV risk management in patients suffering from RA. We excluded animal studies and articles that did not discuss the management and prevention of CV risk. We followed the Preferred Reporting Items for Systematic Reviews and Meta-Analyses (PRISMA) 2020 guidelines for the review (Figure 1). Only the data collected from published papers was reviewed, eliminating the need for ethical approval.

Systematic Literature Search and Study Selection

A thorough search was conducted using Google Scholar and PubMed, including Medline. We also searched for studies mentioned in editorials, review articles, and commentaries on PubMed, as well as additional studies that met our inclusion criteria.

Using specific criteria, we independently reviewed a list of abstracts for inclusion. We included studies that focused on CV risk management in patients suffering from RA. Animal studies were excluded (Table 1). Four reviewers conducted a dual review, and disagreements were resolved through discussion. We established specific criteria for including and excluding participants to achieve our study goals.

**Table 1:** Criteria adopted during the literature search process.

Inclusion criteria	Exclusion criteria
Human studies	Animal studies
Age ≥19 years	Age ≤19 years
English text	Non-English text
Articles published since 2013	Articles published before 2013
Male and female gender	Patients without rheumatoid arthritis
Free, full-text papers	

The population, intervention/condition, control/comparison, and outcome (PICO) criteria were used to conduct a thorough literature review on databases such as PubMed (including Medline) and Google Scholar Libraries (Table 2).

**Table 2:** Search strategy, search engines used, and the number of results.

Database	Search strategy	Search engine
PubMed	Prevention of cardiovascular disease in rheumatoid arthritis	27
	Cardiovascular risk management in rheumatoid arthritis	11
	Management of cardiovascular complications in rheumatoid arthritis	06
Google Scholar	Cardiovascular risk management in rheumatoid arthritis	20,200
	Prevention of cardiovascular disease in rheumatoid arthritis	23,500
	Cardiovascular complications management in rheumatoid arthritis	19,300

The Medical Subject Headings (MeSH) approach was utilized to create a comprehensive search strategy. Filters were applied, as shown in Table 3.

**Table 3:** Search strategy and filters used for the literature search.

Database	Search Strategy	Results
PubMed	((((rheumatoid arthritis[Title/Abstract]) AND (cardiovascular risk management in rheumatoid arthritis[Title/Abstract])) OR (heart complications in rheumatoid arthritis[MeSH Terms])) OR (rheumatoid factor[MeSH Terms])) AND ((“2013/01/01”[Date - Publication]: “2023/07/01”[Date - Publication]))	34; Filters applied: Article type: clinical trials, meta-analyses, systematic reviews, and randomized control trials; Publication date: 10 years; Species: humans; Article language: English; Sex: female, male
Google Scholar	cardiovascular risk management in rheumatoid arthritis OR management of heart disease associated with rheumatoid arthritis	3,33,000; Filters applied: Publication date: 10 years

Quality Appraisal

Various quality assessment tools were utilized to ensure the reliability of the selected papers. Randomized control trials were evaluated using the Cochrane Bias Risk Assessment Tool. For non-randomized trials and observational studies, we used the Newcastle-Ottawa Tool. We used the PRISMA checklist for systematic reviews. In case of any confusion in the classification, we utilized the Scale for Assessment of Narrative Review Articles (SANRA) to assess the quality of the articles (Table 4).

**Table 4:** Tools used for quality appraisal. PRISMA = Preferred Reporting Items for Systematic Reviews and Meta-Analyses; SANRA = Scale for Assessment of Narrative Review Articles; RCT = randomized controlled trial

Quality approval tools used	Types of studies
Cochrane Bias Tool Assessment	RCTs
Newcastle-Ottawa Tool	Non-RCT and observational studies
PRISMA Checklist	Systematic reviews
SANRA Checklist	Any other studies without a clear methodology selection

Results

After going through the three selected databases, i.e., PubMed, Medline, and Google Scholar, 333,034 articles were extracted, following which we applied specific criteria that excluded 332,432 articles. Out of the remaining 602 papers, we chose not to use 541 because they had unsatisfactory titles and abstracts. We closely studied the remaining 61 papers and excluded 35 as they did not meet our inclusion criteria. Finally, a thorough quality check was conducted on the remaining 26 papers, of which 19 were excluded because either their abstract did not fulfill the demands of the paper or the article did not mention details about the research. Finally, seven papers were included in our final systematic review, and the results are shown in Table 5.

**Table 5:** A summary of the studies. RA = rheumatoid arthritis; CV = cardiovascular; CVD = cardiovascular; QUEST = Questionnaires in Standard Monitoring of Patients; DMARDs = disease-modifying antirheumatic drugs; TNF = tumor necrosis factor; SCORE = Systematic Coronary Risk Evaluation; EULAR = European League Against Rheumatism

Author/Year	Country	Study design	Database used	Conclusions
Zegkos et al., 2016 [1]	Greece	Review article	Google Scholar	CV risk in RA can be managed by lifestyle modification, control of disease activity by the use of conventional and biological DMARDs, and pharmacological control of traditional risk factors by the use of antihypertensives and statins
Naranjo et al., 2019 [4]	15 countries were included in the study: Denmark, Finland, France, Germany, Ireland, Italy, the Netherlands, Poland, Serbia, Spain, Sweden, Turkey, United Kingdom, United States, and Argentina	Questionnaires in Standard monitoring of patients with RA (QUEST)-RA cross-sectional study	Google Scholar	Prolonged use of DMARDs, glucocorticoids, and TNF-alpha blockers is associated with a reduced risk of CV events associated with RA
van den Oever et al., 2013 [8]	The Netherlands	Evidence and expert opinion	Google Scholar	Periodical screening of all RA patients for CV risk at an early stage of the disease, smoking cessation, regular checkups of the lipid profiles, regular blood pressure, and blood sugar assessments can help prevent adverse CV events
Gualtierott et al., 2017 [9]	Italy	Review article	Google Scholar	Screening of high-risk patients using SCORE or EULAR. The choice of therapy should be based on the CV risks of an individual patient. DMARDs can be used to reduce the inflammation. The daily dosage and treatment duration of glucocorticoids should be kept low. A healthy lifestyle and a Mediterranean diet can be useful in the management of CVD in RA patients
Daninger et al., 2014 [10]	Austria	Systematic literature review	PubMed	Statins can be used for the primary prevention of CV events in RA patients. Discontinuation of statins was found to increase the risk of CV mortality in RA patients
Soulaidopoulos et al., 2018 [11]	United Kingdom	Literature search	Google Scholar	The lipid-lowering effect and possible anti-inflammatory and angio-protective effects of statins make them an ideal choice for RA-related CV risk
Jagpal et al., 2016 [12]	United States	Narrative review	Google Scholar	A multidisciplinary approach is required to improve CV outcomes and decrease CV mortality among RA patients. CV risk assessment and management of the traditional risk factors are important aspects of reducing CV events in RA patients. Other factors that should be considered are hypertension, insulin resistance, body weight, smoking, lipid profile, and physical activity

Discussion

Regarding CV risk management in RA patients, the first step is lifestyle modification. Keys for successive treatment of CV events in RA suggested by rheumatologists are to (1) stop smoking and (2) get physically fit. The next important step is the determination of the CV risk profile, including an assessment of blood pressure and lipid profile. Using calculators such as Framingham and SCORE, the 10-year CV risk in RA patients can be calculated [8]. However, the presence of traditional risk factors such as hypertension, smoking, dyslipidemia, and obesity cannot fully explain the high magnitude of CVDs in patients with RA [1].

Use of Drugs in Lowering CV Events in RA Patients

Methotrexate is found to lower the CV risk in patients with RA compared to patients who have never received disease-modifying anti-rheumatic drugs (DMARDs) [4]. Methotrexate is found to elevate homocysteine levels, a folic acid antagonist, and may interfere with homocysteine metabolism; hence, dietary supplementation with folic acid is recommended in patients receiving methotrexate [9]. Although some studies have shown an increased risk of CV events with prolonged use of glucocorticoids, others found no association between CV events and glucocorticoid exposure. On the contrary, long-term use of glucocorticoids was independently associated with a reduced risk of cardiovascular events in patients with RA [4]. Nevertheless, non-steroidal anti-inflammatory drugs (NSAIDs) and glucocorticoids should be used with caution in RA patients. Among NSAIDs, diclofenac and ibuprofen are particularly contraindicated in patients with documented CVD [9].

The high-grade inflammatory state of the disease has been linked to the development of premature atherosclerosis. TNF-alpha is an inflammatory cytokine released by activated monocytes, macrophages, and T lymphocytes. This promotes the inflammatory response and causes dyslipidemia and insulin resistance in RA patients. Therefore, TNF-alpha inhibitors are found to lower the potential risk of inflammation [7,13]. However, it has been found that TNF-alpha is associated with higher blood pressure as it may cause vascular damage by inducing inflammation and causing vascular damage via oxidative stress [1].

Statins were associated with reduced CV events in RA patients. Statin discontinuation is associated with an increased risk of acute myocardial infarction and CV mortality in RA patients [11].

Dyslipidemia

High levels of total cholesterol and low-density lipoprotein (LDL) and low levels of high-density lipoprotein (HDL) are found in RA patients. All these changes result in an unfavorable atherogenic profile [1]. Statins can be used in the primary prevention of CV events in RA patients. Statin discontinuation is associated with an increased risk of acute myocardial infarction and CV mortality in RA patients [10]. However, some studies do not support the effectiveness of statins in patients with chronic diseases such as RA [14]. Low doses of atorvastatin at 5-10 mg per day in combination with proper control of chronic inflammation were found to be significantly effective in lowering lipid levels [11]. For primary prevention, treatment with statins should be started once the LDL cholesterol is above 2.5 mmol/L. For secondary prevention, treatment with 80 mg atorvastatin or simvastatin 20-40 mg was found to be effective in one of the trials [11].

Combination therapy of DMARDS with prednisolone is also found to reduce the atherogenic index (increase in total cholesterol and HDL cholesterol) [11]. CV risk is underestimated even when the EULAR correction is applied. In such cases, the detection of plaque by carotid ultrasound can be useful in predicting CV risk when the SCORE assessment or EULAR correction is around the threshold [9]. Arterial stiffness may be assessed with aortic pulse wave velocity and augmentation index. They are considered independent markers of CV events but the vascular morphology assessed by these markers is impaired in RA [11].

There is an increased prevalence of insulin resistance in RA patients due to lipid metabolism dysregulation. Other factors include abdominal obesity, the use of antihypertensive drugs, and the use of glucocorticoids in the alteration of glucose metabolism in RA [15]. Insulin resistance can lead to several atherogenic processes. Long-term positive effects are observed with the use of TNF-alpha antagonists, infliximab, and etanercept on insulin resistance in RA patients [1].

Although there is a very high prevalence of hypertension in RA patients, it is underdiagnosed and treated suboptimally. A study found that a 20 mm increase in systolic blood pressure in RA patients is associated with 1,572 additional ischemic heart events yearly [12]. Antihypertensives should be started only if the systolic blood pressure is above 140 mmHg [8].

RA patients are more physically inactive compared to non-RA patients. Hence, there is a higher mean index of body mass measurement and waist circumference [16]. Any energy expenditure due to physical activity is strongly protective against CVD morbidity and mortality. Physical activity is found to have beneficial effects on CV risk reduction, but the major concern is that many patients do not reach sufficient levels of physical activity. Hence, RA patients should be encouraged to perform physical activity [17].

RA patients are more likely to smoke compared to non-RA patients [1,12,16]. It has been found that cigarette smoking is associated with increased disease severity and poor response to treatment. Therefore, smoking cessation becomes an important part of reducing CV adverse events in RA patients [8,12].

Limitations

One potential limitation of our study is the exclusion of non-English-language papers which may result in missing relevant data from other regions or populations. We also restricted our study to articles published in the last 10 years. While this ensures the inclusion of recent research, it may overlook valuable insights from older studies.

## Conclusions

CV risk management requires the efforts of both a responsible doctor and the patient. Certain efforts on the part of patients are required, such as smoking cessation and regular checkups of blood pressure, blood sugar levels, and lipid profiles. Tight control of RA will lower the risk of CV events in these patients. Statins and antihypertensives are necessary only if the calculated CV risk is above a certain threshold. DMARDs have shown promising results in lowering adverse CV events by controlling disease activity in RA patients. Management of dyslipidemia should be considered a beneficial component for reducing CV risk. Annual CV screening of all RA patients can reduce the risk of CV events. Early diagnosis and progressive management of RA may contribute to the control of disease activity. All patients should be educated about a healthy lifestyle, the importance of exercise, and smoking cessation, regardless of their risk levels.
